# Health service utilization in IBD: comparison of self-report and administrative data

**DOI:** 10.1186/1472-6963-11-137

**Published:** 2011-05-31

**Authors:** Teresa Longobardi, John R Walker, Lesley A Graff, Charles N Bernstein

**Affiliations:** 1 University of Manitoba IBD Clinical and Research Centre, 715 McDermot Avenue, Winnipeg, R3E 3P4, Canada; 2 Department of Internal Medicine, University of Manitoba, 715 McDermot Avenue, Winnipeg, R3E 3P4, Canada; 3 Department of Clinical Health Psychology, University of Manitoba, 715 McDermot Avenue, Winnipeg, R3E 3P4, Canada; 4 Department of Finance, University of Puerto Rico - Rio Piedras, Edificio Ana Maria O'Neill, San Juan, 00931, Puerto Rico

**Keywords:** Self-report, administrative data, health service utilization, inflammatory bowel disease, Crohn's disease, ulcerative colitis

## Abstract

**Background:**

The reliability of self-report regarding health care utilization in inflammatory bowel disease (IBD) is unknown. If proven reliable, it could help justify self-report as a means of determining health care utilization and associated costs.

**Methods:**

The Manitoba IBD Cohort Study is a population-based longitudinal study of participants diagnosed within 7 years of enrollment. Health care utilization was assessed through standardized interview. Participants (n = 352) reported the total number of nights hospitalized, frequency of physician contacts in the prior 12 months and whether the medical contacts were for IBD-related reasons or not. Reports of recent antibiotic use were also recorded. Actual utilization was drawn from the administrative database of Manitoba Health, the single comprehensive provincial health insurer.

**Results:**

According to the administrative data, 15% of respondents had an overnight hospitalization, while 10% had an IBD-related hospitalization. Self-report concordance was highly sensitive (92%; 82%) and specific (96%; 97%, respectively). 97% of participants had contact with a physician in the previous year, and 69% had IBD-related visits. Physician visits were significantly under-reported and there was a trend to over-report the number of nights in hospital.

**Conclusions:**

Self-report data can be helpful in evaluating health service utilization, provided that the researcher is aware of the systematic sources of bias. Outpatient visits are well identified by self-report. The discordance for the type of outpatient visit may be either a weakness of self-report or a flaw in diagnosis coding of the administrative data. If administrative data are not available, self-report information may be a cost-effective alternative, particularly for hospitalizations.

## Background

It is estimated that 0.6% of Canadians and 0.4% of Americans have Crohn's disease (CD) or ulcerative colitis (UC), the two forms of inflammatory bowel disease (IBD) [1,2]. These chronic diseases are characterized by episodic flares that can contribute to significant disruption and overall poor quality of life. With the median incident age between 20 and 30 years, patients are often required to take medication for the duration of their lives. Up to one-third of UC and almost three-quarters of CD patients will require surgery in their lifetime [3,4]. Health care utilization for IBD is higher than in the general population [5].

There is interest in understanding heath care utilization in IBD as it informs cost-effectiveness analysis and resource allocation planning, including decisions about adopting expensive medications and diagnostic tests. One context is determining base rates of health care utilization and then the impact of any intervention at reducing them. The two most widely used methods of data collection for health service utilization are administrative records and self-report [6,7]. Self-report measures are widely used in economic and epidemiological research for representative population samples [6]. There have been two large-scale reviews comparing administrative to self-report data [6,7]. Each method has advantages and disadvantages. Health insurance claims records are a commonly used form of administrative data. However, these data are costly to maintain and analyze. Privacy and administrative issues limit access by independent researchers. Self-report data, while less costly to collect, may be inaccurate due to poor recall, particularly when longer time periods are considered or more complex categorization of information is needed [6-8]. Previous studies of self-report data on health care utilization suggest that concordance with administrative data, depends on the level of cognition and age of respondents, the severity of illness, length of recall period, and types of utilization assessed [6,7]. Given that errors in reporting have been found to be associated with some disease groups, and since IBD patients have not been among the study populations to date, careful examination of concordance in an IBD sample is needed to establish accuracy [8]. This study directly compared self-report and administrative data regarding hospital and physician visits over the prior 12 months for a population-based IBD cohort. We were interested in evaluating the accuracy and limitations of self-report data.

## Methods

This study was approved by the University of Manitoba Research Ethics Board and the Manitoba Health-Health Information Privacy Committee.

### Data Sources

#### Administrative Data

In this province of 1.15 million, Manitoba Health is the government organization that serves as the single, comprehensive health insurance provider for all residents [9]. The organization maintains four large databases. These include a population registry, hospital discharge database, physician claims database and a prescription drug purchases database. The population registry contains a unique personal health identification number for each insured individual which is used to link the databases. This system is described in detail on the Manitoba Centre for Health Policy Website [10]. The accuracy of the provincial administrative health data has been demonstrated across a number of studies for various medical conditions [10-14].

The methods used to establish and validate an administrative definition of IBD in the provincial database were described by Bernstein and colleagues, and the IBD identification algorithm was found to have sensitivity and specificity of 90% [5,15]. In the 2005-2006 fiscal year, there were 7375 IBD cases identified in the population registry, which were subsequently included in an epidemiology database. All individuals in this University of Manitoba IBD Epidemiology Database were mailed invitations to enroll in the University of Manitoba IBD Research Registry, a database used by our Centre, and almost half of the province's IBD population agreed to participate [15].

### Manitoba IBD Cohort

The Manitoba IBD Cohort Study is a prospective longitudinal study following adults with IBD who were diagnosed within 7 years of enrollment [16]. This population-based study was initiated in 2002, recruiting eligible participants from the University of Manitoba IBD Research Registry. It has collected information on disease course, health service utilization, and mental and physical health outcomes. Participants are surveyed every 6 months, and interviewed annually. There were 388 individuals enrolled in the Manitoba IBD Cohort Study. At the time health care utilization information was obtained (12-month interview), 352 cohort study participants had complete data, were insured by Manitoba Health, and had their IBD diagnosis independently confirmed by chart review.

Demographic and disease variables included age and disease duration as of the date of interview, sex, disease type (CD or UC), and disease activity in the prior 12 months. Disease activity was assessed based on the Manitoba IBD Index (MIBDI), a validated patient report measure of symptom frequency over a six-month period [17]. Disease activity across the 12 months was categorized as consistently inactive, consistently active, or fluctuating between inactive and active, based on two consecutive six-month MIBDI assessments.

### Assessment of Health Care Utilization: Self-report

Data on health care utilization were obtained from the Cohort participants through standardized interview between the months of August 2003 and January 2005, the time period during which the Cohort 12-month follow up interviews took place. The questions were drawn directly from a large national health survey (Canadian Community Health Survey) [18]. Respondents were told to consider the prior 12-month period. They were asked whether they had been a patient overnight in a hospital (excluding emergency room visits) and if so, how many nights they spent in hospital over the 12-month recall period. They were also asked how many nights were for IBD reasons. In addition, they were asked whether they had seen a general or specialist physician, whether they had seen a physician for IBD reasons and how many physician visits they had. Frequency of physician visits for IBD reasons was not recorded.

Participants brought medication used in the prior 2 weeks to the interview. Antibiotics were selected for comparison of self-report and administrative data as they are typically consumed soon after purchase, with little discretionary use by the patient. Other drugs may have been purchased outside the 2-week window but consumed during the recall period leading to a false positive or alternately, purchased during the recall period, but consumed at another time resulting in a false negative. Seven types of antibiotics were tracked: amoxicillin, ciprofloxacin, clarithromycin, clindamycin, erythromycin, metronidazole, and penicillin.

### Assessment of Concordance Between Administrative Data and Self-Report

The self-report data regarding utilization was linked to the administrative databases, using the interview date to extract utilization records from the administrative data for the previous 12 months. Variables for any overnight hospitalization (dichotomous) and the total number of nights in hospital were created. An IBD-related overnight hospitalization was flagged if any one of the hospital diagnosis codes had an ICD-9-CM prefix of 555 (Crohn's disease) or 556 (ulcerative colitis), or since April 1, 2004, ICD-10-CM code prefix of K50 or K51. A physician visit was flagged if a physician consultation occurred at a time period outside of hospitalization. ICD-9-CM physician codes of 555 (CD) and 556 (UC) were used to create variables for the total number of physician visits and to indicate that an IBD-related visit occurred.

Sensitivity and specificity measures were computed to summarize concordance of self-reported use with administrative data. To test the concordance in the self-reported frequency of use against the administrative data, the concordance correlation coefficient was computed and the correlated t-test was used to compare the means of the distributions [19,20]. The concordance correlation coefficient is the multiple of the Pearson correlation coefficient, which is a measure of precision (how far each observation deviates from the best-fit line), and the bias correction factor, which is a measure of accuracy (how far it deviates from the 45° line). One-way analysis of variance and Bonferroni multiple comparisons were used to detect differences in frequency of physician contacts or self-reported contacts attributable to the level of disease activity.

### Representativeness of the Manitoba IBD Cohort Participants

To assess how well the Manitoba IBD Cohort participants modeled the broader population in the province with IBD, they were compared on demographic characteristics to a population reference group comprised of all individuals in the IBD Epidemiology Database who (a) were also in the population registry file over the period of the Cohort Study 12-month interviews from August 6, 2003 to January 24, 2005, (b) had IBD of similar duration as the Manitoba IBD Cohort sample, (i.e., 2 to 10 years at the end of the period January 24, 2005), and (c) were not already participating in the Manitoba IBD Cohort Study. A total of 2963 patients from the University of Manitoba IBD Epidemiology Database met these criteria and formed the population reference group. For the purposes of standardization in comparison, age was computed as of January 24, 2005 for all the Manitoba IBD Cohort and University of Manitoba IBD Epidemiology Database subjects. Proportions were compared using Chi-square tests. STATA SE 10.1 (College Station, Texas) was used to conduct all of the analyses.

## Results

### Participant Characteristics and Representativeness

Table 1 describes the demographic characteristics and the health care utilization of the Manitoba IBD Cohort Study participants and the population reference group from the University of Manitoba IBD Epidemiology Database from August 6, 2003 to January 24, 2005. Although the reference period for testing individual concordance in administrative data and self-report was 12 months for health service utilization, and 2 weeks for antibiotic prescriptions, this 18-month period was used to compare Cohort Study respondents against IBD subjects in the administrative data as it coincided with the total period over which the 12-month Cohort study interviews were conducted. The Cohort group did not differ significantly on age, sex, or proportion of disease subtype (CD or UC) from the reference group, suggesting excellent representativeness of the Cohort. Manitoba IBD Cohort participants had higher levels of health service use over this period, and were also more likely to use services for IBD reasons.

**Table 1:** Demographic Characteristics and Health Service Utilization Comparing IBD Cohort Participants and a Population Reference Group from the IBD Epidemiology Database Across an 18-Month Period by Crohn's Disease (CD) and Ulcerative Colitis (UC)

	Cohort Study	Epidemiology Database	Statistical Comparison
Variable	n = 352	n = 2963	**p-value**^**§**^
Age < 40	43%	40%	NS
Female	60%	56%	NS
CD (UC)	53% (47%)	48% (52%)	NS
Hospitalized Overnight (CD | UC)	18% (21% | 16%)	18% (20% | 15%)	NS
Hospitalized Overnight for IBD (CD | UC)	9% (12% | 6%)	6% (9% | 3%)	p = 0.03 (p = 0.19 | p = 0.04)
Physician Visit (CD | UC)	99% (99% | 99%)	85% (84% | 85%)	p < 0.0001 (p < 0.0001 | p < 0.0001)
Physician Visit for IBD (CD | UC)	75% (80% | 70%)	44% (45% | 43%)	p < 0.0001 (p < 0.0001 | p < 0.0001)
Any Prescription (CD | UC)	96% (96% | 96%)	79% (79% | 83%)	p < 0.0001 (p < 0.0001 | p < 0.0001)
Prescription for Antibiotic (CD | UC)	54% (63% | 44%)	46% (47% | 46%)	p = 0.0045 (p < 0.0001 | p = 0.62)

**Note: The Manitoba IBD Cohort study participants provided self-report and administrative data concerning health care utilization. The University of Manitoba IBD Epidemiology Database included all validated IBD cases in the provincial administrative database (not participating in the Cohort Study) with similar disease duration (2 to 10 years) to the Cohort participants**.

**^§ ^Chi-square test of independence was used**.

More detailed clinical information was obtained from the Cohort participants. Twenty-three percent had consistently inactive disease over the previous 12 months, 21% had fluctuating disease and 56% had consistently active disease. Disease activity patterns across time were similar between those with CD and UC.

### Concordance of Self-report Compared to Administrative Health Care Utilization Data

The comparisons of self-report and administrative measures of health service utilization over the 12 months prior to the interview are shown in Table 2. The administrative data indicated that 52 out of 352 (15%) of Manitoba IBD Cohort respondents were hospitalized overnight and 34 out of 352 (10%) were hospitalized overnight for IBD reasons. There was reasonably good agreement of self-report data with high sensitivity and specificity. Taking the administrative data as the reference group, there were 4 and 6 false negatives respectively for both non-IBD-related and IBD-related hospitalizations, 11 false positives for non-IBD-related reasons, and 10 false positives for IBD-related reasons.

**Table 2:** Sensitivity and Specificity of Self-report Compared to Administrative Data for Overnight Hospitalization and Physician Visits (*n *= 352)

Hospitalization		Admin Data			Sensitivity	Specificity
		Yes	No	Subtotal		
Self-report	Yes	48	11	59	92%	96%
	No	4	289	293		
	Subtotal	52	300			
						

**Hospitalization**		**Admin Data**			**Sensitivity**	**Specificity**
**IBD-related**		Yes	No	**Subtotal**		
**Self-report**	Yes	28	10	**38**	82%	97%
	No	6	308	**314**		
	**Subtotal**	**34**	**318**			

						

**Physician Visit**		**Admin Data**			**Sensitivity**	**Specificity**
		Yes	No	**Subtotal**		
**Self-report**	Yes	336	5	**341**	98%	50%
	No	6	5	**11**		
	**Subtotal**	**342**	**10**			

						

**Physician Visit IBD-related**		**Admin Data**			**Sensitivity**	**Specificity**
		Yes	No	**Subtotal**		
**Self-report**	Yes	213	40	**253**	88%	63%
	No	30	69	**99**		
	**Subtotal**	**243**	**109**			

To clarify discrepancies for hospitalizations, specific cases were examined more closely. Errors included the failure to estimate a stay within the recall period, omitting stays following an emergency room visit, mistakenly including day surgery as hospitalization, and misclassifying an IBD stay. Of the four cases in which overnight hospitalization was not reported but was evident in the administrative database, two were seen by a physician in an emergency room and were subsequently hospitalized. As participants were told not to report emergency room visits, some may have failed to report a hospitalization that was a consequence of an emergency room visit, potentially accounting for these two discrepancies. Further, another one of these cases had a 10-night stay in hospital, which occurred just less than a year before the interview. Thus, an explanation for failure to report a hospitalization may have been the long recall period leading the patient to consider the visit as outside the specified period.

Eleven respondents reported a hospitalization when there was no record of one in the administrative database. A closer review of the administrative record indicated that four of these 11 were hospitalized 15 months to 2 years before their interview date. Four others had been admitted for day surgery within a year of the interview and may have considered this in their reporting.

Considering IBD-related hospitalizations, 10 respondents reported an IBD-related hospitalization that was not congruent with the administrative data. According to the administrative record, one of the 10 had an admission for liver disease. Four did have an IBD-related hospitalization, but it was 15 months to 2 years before the interview date. There was no record of any overnight hospitalization within the province for the other 5 patients. It is certainly possible that some respondents had been hospitalized out of province, but this information was not available. Six respondents did not report an IBD-related hospitalization that was on record in the administrative data. Of those, two had hospitalizations that occurred just under the annual threshold according to the administrative data, and 4 had reported a hospitalization but did not specify that it was an IBD-related stay.

Although overnight hospitalization was uncommon, in contrast, 97% of the Manitoba IBD Cohort participants saw a physician in the previous year, with 69% visiting a physician for IBD-related reasons, as seen in Table 2. Sensitivity for reporting physician visits was similar to sensitivity for reporting hospitalizations, however specificity was weaker likely because so few did not see a physician.

With regard to discrepancies in reporting physician visits, the 6 respondents who had visits recorded in the administrative data but who did not directly report physician visits had a total of 22 visits recorded (range [1,9] visits). The most recent visits before the interview date for these 6 people ranged from 4 to 338 days earlier. Of the 30 who did not report an IBD-related visit, two of them had not reported any visits, and 28 reported visits but did not identify IBD as the reason. In contrast, 40 respondents reported having an IBD-related visit, but it was not coded as such in the administrative data. Of those 40, 37 had a recorded visit, and 3 had no visits recorded in the administrative database. Of the 37 who had a recorded visit, the diagnosis codes showed no clear indication of IBD co-morbidities such as abdominal pain, diarrhea or arthritis although for 9 respondents, the records indicated a code for "other and unspecified noninfectious gastroenteritis and colitis" (ICD-9-CM code 558). For both types of discordant reporting, the discrepancy was primarily in the understanding or recall of the details of the visit, not whether one had occurred.

Table 3 provides more detailed information comparing the number of hospital nights and physician visits based on self-report or administrative data. Considering the number of physician visits, while the self-report and administrative data were significantly correlated, the self-report data underestimated the number of physician visits relative to the administrative data. In analyzing the number of hospital nights, we considered both the means for all respondents and the means for those respondents with at least one hospital admission (based on either administrative data or self-report) considering overall admissions and IBD-related admissions. The administrative and self- report data were highly correlated for the whole group, as well as for the subgroup of respondents with at least one admission. While there was a tendency to over-estimate the number of hospital nights in the self-report data, the differences were not large enough to be statistically significant.

**Table 3:** Comparison of Self-Report and Administrative Data On Number of Physician Visits and Hospital Nights in the Last 12 Months

Variable	Self-report Mean (standard error)	Admin Data Mean (standard error)	*p-*value^§^	Concordance Correlation Coefficient Pearson Correlation Coefficient (Bias Correction Factor C_b_)
All respondents (*n *= 352)				
Physician Visits	6.92 (0.37)	11.09 (0.50)	<0.0001*	0.58^† ^0.68 (0.85)
Hospital Nights	2.20 (0.62)	1.75 (0.42)	0.09	0.86^† ^0.93 (0.93)
Hospital Nights IBD	1.96 (0.61)	1.46 (0.40)	0.13	0.80^† ^0.88 (0.91)
Respondents with at least one hospital admission by either self-report or administrative data				
Hospital Nights *n *= 63	12.29 (3.16)	9.76 (2.07)	0.09	0.84^† ^0.92 (0.91)
Hospital Nights IBD *n *= 44	15.70 (4.44)	11.68 (2.76)	0.13	0.75^† ^0.84 (0.89)

^§^Correlated *t*-test, *statistically significant (p < 0.0001), ^†^statistically different from 0 at 0.05 level of significance.

Note: Maximum number of physician visits for the self-report data was 57 and for administrative data it was 67. Maximum hospital nights for self-report data was 170 and for administrative data it was 91.

At the time of the 12-month Manitoba IBD Cohort interview, 16 of 352 Cohort participants (4.5%) reported they were taking either one or more of seven antibiotics over the preceding two weeks. There was complete (100%) agreement between participants' reported use of antibiotics, as assessed by review of their submitted prescription containers, and the purchase of antibiotics recorded in the database, resulting in 100% sensitivity and specificity (data not shown).

### Disease Activity and Reporting Concordance

Since nearly all of the Manitoba IBD Cohort participants had seen a physician in the previous year, the relationship of disease activity and accuracy of reporting number of physician visits was assessed. As shown in Table 4, based on both self-report and administrative data, those with inactive disease over the 12-month period had significantly fewer visits than those with consistently active disease, by almost half (self-report: F = 8.31, p = 0.0003; administrative data: F = 8.46, p = 0.0003). Regardless of whether disease was inactive, fluctuating or active across the full 12 months, the number of physician visits tended to be underestimated by at least one-third, comparing the self-report information to the administrative data (p < 0.0001).

**Table 4:** Comparison of self-report and administrative data on number of physician visits by levels of disease activity

Disease Activity	Self-report Mean (standard error)	Admin Data Mean (standard error)	*p-*value^§^	Concordance Correlation Coefficient Pearson Correlation Coefficient (Bias Correction Factor C_b_)
Consistently inactive (*n *= 77)	4.42^a ^(0.45)	7.75^b ^(0.83)	<0.0001	0.455^† ^0.629 (0.724)
Fluctuating (*n *= 71)	6.83 (0.78)	10.41 (0.91)	<0.0001	0.543^† ^0.619 (0.877)
Consistently active (*n *= 186)	8.24 (0.58)	12.88 (0.78)	<0.0001	0.592^† ^0.692 (0.855)

^§^Correlated *t*-test

^a,b^Consistently inactive and consistently active are statistically significantly different (p < 0.0001) by one-way ANOVA and Bonferroni multiple comparisons.

## Discussion

An advantage of the sample used in this study was that it was community-based from the population of patients in Manitoba with IBD, and not from those attending health care settings for treatment [16]. Further, the Manitoba IBD Cohort sample was found to be closely representative of the province's IBD population, based on demographic comparisons. Interestingly, the Cohort participants were more likely to use health care services and take prescription medication than the general IBD population. It may be that persons who are experiencing more symptoms, or are more engaged in the health care system are more likely to agree to take part in a longitudinal study. From the current study we were unable to determine if the increased rate of utilization was due to legitimate medical illness. Alternatively, participation in a study may increase health care utilization due to improved rapport with physicians as suggested in a report of persons enrolled in a study of traumatic events where higher rates of health care utilization was found following 12-month enrolment in a research study [21].

Data sets contain both random and systematic sources of error. Considering self-report and administrative health service utilization data, random sources of error regarding service type and frequency of use may be related to problems in memory (for self-report data), data entry errors (administrative data), and interpretation or coding differences (both self-report and administrative data). For example, a patient may have understood a visit for a skin rash due to IBD medication side effects to be an IBD-related visit, whereas the physician coded the visit for dermatitis. In this study, when discrepancies between self-report and administrative data were reviewed in more detail, some of these types of errors were identified, including recall errors (i.e., reporting a hospitalization that was outside the one year recall period) and likely mis-categorization (e.g., emergency room visits reported as hospitalizations). Discrepancies related to emergency room visits have been identified in other studies [22,23]. Unfortunately we were unable to verify this directly in our sample, since emergency room visits are not recorded in the administrative data.

Systematic errors, on the other hand, may be identified by comparing one imperfect source of information (such as self-report data) with another imperfect but probably more accurate source of information (such as administrative data), and looking for directional trends when there are discrepancies. The administrative health data have been evaluated and established as a reliable source of health care utilization information [10-14]. The perfect match of patient and administrative data for the medication use found in this study, which used a short time frame, and a specifically defined health service (i.e. antibiotic dispensing) provides further confidence in the reliability of the data. Comparison of self-report and administrative data in this sample suggested that there were systematic sources of error concerning length of hospitalization and number of physician visits over a one-year recall period. There was a trend to over-report, by 25% to 35%, the number of nights in hospital, regardless of whether the hospitalization was for IBD or other reasons. While not statistically significant in this sample a difference of this magnitude would likely have been significant in a larger sample with a higher base rate of hospitalizations. Other studies have found severity of illness associated with over-reporting the length of hospital stays [24]. On the other hand, physician visits, which had a high base rate, were underestimated by 35% to 45% using self-report relative to administrative information, and these discrepancies held across different levels of disease activity. This tendency to under-report physician visits was also found in other studies [6,24]. Researchers should be aware of these systematic errors in reports of hospitalization and physician visit data when evaluating self-report data. The magnitude of these self-report errors will depend on a variety of factors including time, the type of health service utilization reported (including its salience in memory), the medical condition, and characteristics of the specific sample. For instance, the detailed evaluation in this study suggested that several of the discrepancies in reporting may have been avoided with a shorter recall time frame than the 12-month period.

In spite of the clear problems with over-reporting of hospital nights and under-reporting of physician visits, as in previous research there were still relatively high correlations between self-report and administrative data for hospital nights and physician visits [6]. When administrative data are not available, self-report data may still be able to provide general estimates of health care use, although further attention could be paid to methods to enhance accuracy.

In their review of accuracy of self-report information concerning health care utilization, Bhandari and Wagner identified some modifiable factors that affect accuracy of this type of information such as questionnaire design, mode of data collection, and memory aids [6]. Recent research suggests that memory aids and probes using approaches like the event history calendar can facilitate better accuracy and substantially reduce under-reporting of health care utilization, including when longer time intervals (such as 12-month periods) are used [25,26]. Recalling the sequence of health care events (for example: Who did you see first? And then whom did you see after that?) can help the respondent recall more specific details than using just a single question. In IBD, for instance, some services (such as endoscopy) are more likely so inquiring specifically about those services may assist in more accurately reporting utilization. In work with an elderly population, one group obtained very accurate self-report data by instructing participants in a longitudinal study to track health utilization via a calendar, and there were regular bi-monthly phone contacts to gather data [27].

As the use of electronic health records becomes more widespread, there is the potential to improve both administrative and self-report data [28]. A growing trend in many countries is to encourage patients to interact with their health information through the use of electronic personal health records. Examples include patient generated health and lifestyle records that are stored and managed using personal computer or web applications, and passive access to provider held records through waiting room kiosks, the internet, or digital copy (such as on a CD or smart card) [29]. These electronic health records can assist patients in more actively managing their health condition through individualized care plans, graphing of symptoms, passive feedback, tailored instructive or motivational feedback, decisional aids, and reminders [29]. Our findings suggest that IBD patients, unaided by tracking prompts such as personal health records or visit information tools such as personal health records or visit receipts, can readily recall medical contacts, but are poor at detailing the specifics such as frequency of physician visits and length of hospital stays. Until high-quality information tools become accessible to patients, when health care utilization is of interest, patients should be encouraged to keep a record of symptoms, utilization and treatments in a diary or in a calendar and not to rely solely on self-recall.

## Conclusions

In summary, the validity of self-report data, as it informs the estimation of health care costs, is critical. This study has drawn attention to some areas of reasonable accuracy and others of systematic inaccuracy in self-report data describing service utilization over a 12-month period. Although recall of a medical contact was good, there were systematic errors in the reported frequency of physician visits (under-reported) and hospital nights (over-reported). If adequate administrative data are not available, self-report data may be a cost-effective alternative, depending on the level and precision of data needed. Use of data collection techniques designed to improve the quality of self-report data may also enhance utility. Recall accuracy may be improved by assessing shorter time frames with more specific time cues and questions about use of specific types of service. Prompts may be used to clarify common areas of miscategorization, such as recall of emergency room visits or day surgeries. Self-report questions may allow opportunities to explore aspects of health care utilization not captured in administrative data.

## Abbreviations

IBD: inflammatory bowel disease; CD: Crohn's disease; UC: ulcerative colitis; ICD-9-CM: International Classification of Diseases Coordination and Maintenance Committee

## Competing interests

In the past 5 years Dr Bernstein has served as consultant or on advisory board of Abbott Canada, Shire Canada, Axcan Pharma, Astra Zeneca Canada, and Janssen Canada and has received an unrestricted educational grant from Axcan Pharma, an unrestricted research grant from UCB Pharma Canada and a research grant from Abbott Canada.

## Authors' contributions

TL contributed to study conception and design, acquisition of data, data analysis and interpretation, drafting of the manuscript and revisions, provided critically important intellectual content, edited and approved the final manuscript. JRW contributed to study conception and design, data analysis and interpretation, revising the manuscript critically for important intellectual content, edited and approved the final manuscript. LAG contributed to study conception and design, data analysis and interpretation, revising the manuscript critically for important intellectual content, edited and approved the final manuscript. CNB contributed to study conception and design, acquisition of data, data analysis and interpretation, revising the manuscript critically for important intellectual content, responded to editors requested revisions, edited the manuscript accordingly, edited and approved the final manuscript. All authors read and approved the final manuscript.

## Pre-publication history

The pre-publication history for this paper can be accessed here:

http://www.biomedcentral.com/1472-6963/11/137/prepub

## References

[B1] Crohn's and Colitis Foundation of Canada The Burden of IBD in Canada 2008 Toronto: The Foundation http://www.ccfc.ca/English/images/proclamations/BIBDC%20FINAL%20October%2029th%20EN.pdf Available at: Accessed June 14, 2009

[B2] Kappelman MD Rifas-Shiman SL Leinman K Kappelman MD Rifas-Shiman SL Kleinman K Ollendorf D Bousvaros A Grand RJ Finkelstein JA The prevalence and geographic distribution of Crohn's disease and ulcerative colitis in the United States Clin Gastroenterol Hepatol 2007 5 1424 9 10.1016/j.cgh.2007.07.012 17904915

[B3] Hendriksen C Kreiner S Binder V Long term prognosis in ulcerative colitis - based on results from a regional patient group from the county of Copenhagen Gut 1985 26 158 63 10.1136/gut.26.2.158 3967834 PMC1432417

[B4] Bernell O Lapidus A Hellers G Risk factors for surgery and recurrence in 907 patients with primary ileocaecal Crohn's disease Br J Surg 2000 87 1697 701 10.1046/j.1365-2168.2000.01589.x 11122187

[B5] Longobardi T Bernstein CN Health care resource utilization in inflammatory bowel disease Clin Gastroenterol Hepatol 2006 4 731 43 10.1016/j.cgh.2006.02.013 16631415

[B6] Bhandari A Wagner T Self-reported utilization of health care services: improving measurement and accuracy Med Care Res Rev 2006 63 217 35 10.1177/1077558705285298 16595412

[B7] Evans C Crawford B Patient self-reports in pharmacoeconomic studies. Their use and impact on study validity Pharmacoeconomics 1999 15 241 56 10.2165/00019053-199915030-00004 10537432

[B8] Wolinsky FD Miller TR An H Geweke JF Wallace RB Wright KB Chrischilles EA Liu L Pavlik CB Cook EA Ohsfeldt RL Richardson KK Rosenthal GE Hospital episodes and physician visits: the concordance between self-reports and medicare claims Med Care 2007 45 300 7 10.1097/01.mlr.0000254576.26353.09 17496713 PMC1904836

[B9] http://www.gov.mb.ca/contact/faq.html#population Information on Manitoba available at: Accessed June 10, 2009

[B10] http://umanitoba.ca/faculties/medicine/units/community_health_sciences/departmental_units/mchp/resources/repository/index.html Accessed March 22, 2011

[B11] Roos LL Mustard CA Nicol JP Registries and administrative data: organization and accuracy Med Care 1993 31 201 12 10.1097/00005650-199303000-00002 8450678

[B12] Roos LL Gupta S Soodeen RA Jebamani L Data quality in an information-rich environment: Canada as an example Can J Aging 2005 24 Suppl 1 153 70 16080132 10.1353/cja.2005.0055

[B13] Kozyrskyj AL Mustard CA Validation of an electronic, population-based prescription database Ann Pharmacother 1998 32 1152 7 10.1345/aph.18117 9825079

[B14] Roos LL Nicol JP A research registry: uses, development, and accuracy J Clin Epidemiol 1999 52 39 47 10.1016/S0895-4356(98)00126-7 9973072

[B15] Bernstein CN Blanchard JF Rawsthorne P Wajda A Epidemiology of Crohn's Disease and Ulcerative Colitis in a central Canadian province: A population-based study Am J Epidemiol 1999 149 10 916 24 10342800 10.1093/oxfordjournals.aje.a009735

[B16] Graff LA Walker JR Lix L Rawsthorne P Rogala L Miller N Jakul L McPhail C Ediger J Bernstein CN The relationship of inflammatory bowel disease type and activity to psychological functioning and quality of life Clin Gastroenterol Hepatol 2006 4 1491 1501 10.1016/j.cgh.2006.09.027 17162241

[B17] Clara I Lix LM Walker JR Graff LA Miller N Rogala L Rawsthorne P Bernstein CN The Manitoba IBD Index: evidence for a new and simple indicator of IBD activity Am J Gastroenterol 2009 104 1754 63 10.1038/ajg.2009.197 19455122

[B18] Gravel R Beland Y The Canadian Community Health Survey: mental health and wellbeing Can J Psychiatry 2005 50 573 9 16276847 10.1177/070674370505001002

[B19] Lin LI-K A concordance correlation coefficient to evaluate reproducibility Biometrics 1989 45 255 68 10.2307/2532051 2720055

[B20] Lin LI-K A note on the concordance correlation coefficient Biometrics 2000 56 324 5

[B21] Sansone RA Sansone LA Wiederman MW Increased health care utilization as a function of participation in trauma research Am J Psychiatry 1997 154 1025 7 9210759 10.1176/ajp.154.7.1025

[B22] Ritter PL Stewart AL Kaymaz H Sobel DS Block DA Lorig KR Self-reports of health care utilization compared to provider records J Clin Epidemiol 2001 54 136 41 10.1016/S0895-4356(00)00261-4 11166528

[B23] Roberts RO Bergstralh EJ Schmidt L Jacobsen SJ Comparison of self-reported and medical record health care utilization measures J Clin Epidemiol 1996 49 989 95 10.1016/0895-4356(96)00143-6 8780606

[B24] Wolinsky FD Miller TR An H Geweke JF Wallace RB Wright KB Chrischilles EA Liu L Pavlik CB Cook EA Ohsfeldt RL Richardson KK Rosenthal GE Hospital episodes and physician visits: The concordance between self-reports and medical claims Med Care 2007 45 4 300 7 10.1097/01.mlr.0000254576.26353.09 17496713 PMC1904836

[B25] Belli RF The structure of autobiographical memory and the event history calendar: potential improvements in the quality of retrospective reports in surveys Memory 1998 6 383 406 10.1080/741942610 9829098

[B26] Belli RF Smith LM Andreski PM Agrawal S Methodological comparisons between CATI event history calendar and standardized conventional questionnaire instruments Public Opinion Quarterly 2007 71 603 22 10.1093/poq/nfm045

[B27] Dubois MF Raîche M Hébert R Gueye NR Assisted self-report of health-services use showed excellent reliability in a longitudinal study of older adults J Clin Epidemiol 2007 60 10 1040 5 10.1016/j.jclinepi.2006.12.011 17884599

[B28] Blumenthal D Tavenner M The "meaningful use" regulation for electronic health records N Engl J Med 2010 363 6 501 4 10.1056/NEJMp1006114 20647183

[B29] Pagliari C Detmer D Singleton P Potential of electronic health records BMJ 2007 335 7615 330 3 10.1136/bmj.39279.482963.AD 17703042 PMC1949437

